# Power Autonomy Estimation of Low-Power Sensor for Long-Term ECG Monitoring

**DOI:** 10.3390/s22145070

**Published:** 2022-07-06

**Authors:** Klemen Bregar, Tomaž Krištofelc, Matjaž Depolli, Viktor Avbelj, Aleksandra Rashkovska

**Affiliations:** Department of Communication Systems, Jožef Stefan Institute, 1000 Ljubljana, Slovenia; klemen.bregar@ijs.si (K.B.); tomaz.kristofelc@ijs.si (T.K.); matjaz.depolli@ijs.si (M.D.); viktor.avbelj@ijs.si (V.A.)

**Keywords:** Bluetooth Low Energy, ECG, wireless sensor, power consumption, autonomy estimation

## Abstract

The paper analyses the autonomy of a wireless body sensor that continuously measures the potential difference between two proximal electrodes on the skin, primarily used for measuring an electrocardiogram (ECG) when worn on the torso. The sensor is powered by a small rechargeable battery and is designed for extremely low power use. However, the autonomy of the sensor, regarding its power consumption, depends significantly on the measurement quality selection, which directly influences the amount of data transferred. Therefore, we perform an in-depth analysis of the power consumption sources, particularly those connected with the Bluetooth Low Energy (BLE) communication protocol, in order to model and then tune the autonomy of the wireless low-power body sensor for long-term ECG monitoring. Based on the findings, we propose two analytical models for power consumption: one for power consumption estimation in idle mode and the other one for power estimation in active mode. The proposed models are validated with the measured power consumption of the ECG sensor at different ECG sensor settings, such as sampling rate and transmit power. The proposed models show a good fit to the measured power consumption at different ECG sensor sampling rates. This allows for power consumption analysis and sensor autonomy predictions for different sensor settings. Moreover, the results show that the transmit power has a negligible effect on the sensor autonomy in the case of streaming data with high sampling rates. The most energy can be saved by lowering the sampling rate with suitable connection interval and by packing as much data as possible in a single BLE packet.

## 1. Introduction

With the advancement of electronics and constant densification of microchips, the development of small and extremely low-power wireless sensors became possible. The energy consumption of wireless body area network (WBAN) sensors is an important factor to be considered in the design phase. For practical reasons, WBAN sensors are generally powered by small batteries with limited overall size and dimensions. Therefore, the available energy is mostly a very limited resource. The available energy can be increased and device lifetime prolonged using different techniques for energy harvesting from radio frequency (RF) energy, thermal energy, solar energy, and mechanical energy [1].

Different technologies suitable for low-power wireless sensor communications were developed during the past years. In 2003, IEEE defined a technical standard for low-rate wireless personal area networks (LR-WPAN)—IEEE 802.15.4—which focuses on low-cost and low-speed communications enabling the emergence of upper-layer communication protocols such as ZigBee, 6LoWPAN, Thread, WirelessHART, MiWi, ISA100.11a, and others [2,3]. Next, the LoRa Alliance introduced LoRa (Long Range) low-power wide-area network technology enabling long-range (more than 10 km) transmissions with low-power consumption but also very low bit rates and throughput capabilities. Finally, the Bluetooth Special Interest Group (SIG) introduced a backward incompatible extension of the standard Bluetooth technology with Bluetooth Low Energy (BLE), enabling novel applications in health care, fitness, and home entertainment industries.

BLE, a proprietary wireless interface, is often selected mainly due to its general ubiquity in modern personal mobile devices. Unlike all the other previously mentioned communication technologies, BLE is the only one readily available in nearly every new smartphone, tablet or smart watch. The main advantages of using standardized and widely spread technology are great availability, standardized communication interfaces, great amount of support available in the community, etc. Despite the ever-present possibility of creating slightly better wireless communication technology regarding the power efficiency per transferred information, the greater availability in consumer devices, and consequently lower price, outweigh small differences in performance.

One particular example that we focus on in this paper is the energy consumption of a miniature smart wireless ECG sensor, which is an important topic when providing long power autonomy in the scope of a framework for long-term ECG monitoring [4], e.g., to discover rarely occurring anomalies in ECG, which often do not occur within 30- or less-second ECG measurements routinely performed within primary health care. In combination with a ubiquitous smartphone with BLE connectivity and a large storage capacity, the hardware requirements for ECG sensors are minimal, making the device viable for mass production with a relatively low price. We were able to analyze such a device, which was recently introduced into the local health-care system.

Energy consumption in battery-powered wireless sensors is important because of its impact on the sensor’s operating time. Having a model to estimate the energy consumption based on the sensor’s activity patterns and workload can help engineers to develop energy-efficient applications and obtain quick estimations without thorough testing with actual devices. It can also help the end user or technician to obtain a good estimate of the expected device operating time according to the device settings. These are the reasons for energy consumption modeling in wireless sensor networks (WSNs) being an active research topic. Energy consumption models have been proposed and evaluated for various communication protocols in WSNs [5,6,7,8], also including BLE [9,10,11]. However, actual sensor uptime and power consumption are affected by many parameters that are difficult to be generalized to all available connectivity solutions and implementations. Power consumption depends on the actual implementation of the communication solution, such as the physical RF implementation, power management, and the most important factor: the actual software running on a wireless sensor device.

Having in mind the above said, in the paper, we demonstrate how a general model can be put in place and evaluated for a special case of low-power sensor for long-term ECG monitoring. Moreover, in this paper, we also shortly illustrate the task of providing guidelines for further development of the ECG sensor, based both on the experience gathered from real-life usage of the sensor and on the analysis of the production version of the sensor circuitry. The real-life experience was gathered from volunteer users [12] and from health-care centers [13]. In particular, the contributions of the paper can be summarized as follows:

- We perform an in-depth analysis of the power consumption sources, particularly those connected with the BLE communication protocol, in order to model and then tune the autonomy of the given wireless low-power body sensor for long-term ECG monitoring. Power consumption estimations are the basis for the sensor autonomy estimation and we thoroughly analyzed the sensor from this angle.
- Based on the findings, we propose analytical models that enable the power consumption estimations for all wireless operating modes of the ECG sensor. The proposed models are validated with the measured power consumption of the ECG sensor at different ECG sensor settings, such as sampling rate and transmit power.
- The resulting models and autonomy estimations enable the engineers to find the optimal wireless ECG sensor settings to optimize the autonomy as well as the hardware of future sensor revisions, given the possible scenarios of sensor use.

The paper is organized as follows. In the next section, the related work from the area of modeling and analyzing energy consumption models is overviewed. In that section, the wireless body sensor for continuous ECG measurements is described as well. Following this, in Section 3, we present the sensor’s operating modes important for power autonomy estimation, including also energy consumption measurements during those modes. The power consumption models are presented in Section 4. Their validation and sensor autonomy analysis subsequently follow in Section 5. The paper concludes with a critical summary of the presented results, limitations of the work, and recommendations for increased autonomy of wireless sensors.

## 2. Background and Related Work

In this section, the related work regarding energy consumption modeling in wireless sensor networks is initially presented, including models and consumption analysis for various communication protocols, such as LoRaWAN and BLE. After that, we present the background and the related work of the considered wireless ECG sensor, focusing of its design and its intended use.

### 2.1. Energy Consumption Modeling in Wireless Sensor Networks

Energy consumption modeling in WSNs is an active research topic. The authors in [14] have proposed a combination of the coordinated duty cycle algorithm (CDCA) and network coding to reduce the energy consumption and improve the transmission reliability in WBAN. They introduced a mathematical model for energy consumption, which was evaluated and compared with the actual implementation using simulations. The authors in [15] proposed a routing approach based on the energy consumption equalization within the sensor device. They developed both realistic and theoretical models for estimating the energy consumption of their proposed routing algorithms and proved its efficiency in minimizing the overall energy consumption. The authors in [5] proposed an extensive analytical energy consumption model and derived the load-balanced optimal routing configuration that maximizes network lifetime. In [16], the authors proposed a simple energy model for WSNs and demonstrated the significant reduction in energy consumption when limiting the frequency of network synchronization events by reducing the duty cycle. The authors in [6] developed a sensor lifetime energy model and evaluated the effects of the duty cycle on the expected energy consumption. They compared their model with the standard STEM-B model. Their model demonstrates that, by increasing the duty cycle, the energy consumption increases proportionally. These publications proposed energy consumption models for complex WSNs where many sensor devices are, in contrast to BLE connections, connected in multihop topologies.

One of the widespread technologies for low-power WSNs is LoRaWAN, with its unique narrow-band modulation which enables low-power, low-rate and long-range narrow-band communications. The authors in [7] proposed an extensive energy consumption model for LoRaWAN Class A sensor node devices. They evaluated all aspects of device activity and energy consumption during the signal processing, energy consumption while receiving response, energy consumption while in sleep mode, etc. They also considered the microcontroller unit (MCU) frequency as a parameter in the proposed energy consumption model. By using the proposed energy consumption model, they analyzed the impact of all LoRaWAN communication parameters on the energy consumption. They compared the sensor node autonomy for three hypothetical LoRaWAN scenarios. Next, modeling solution comes in [8], where the authors proposed energy consumption models for LoRaWAN Class A devices for current consumption estimation, device lifetime estimation, and energy cost of data delivery estimation. The models were developed based on measurements made using the actual LoRaWAN devices. The authors in [17] proposed a simple battery lifetime prediction model based on the measurements for LoRaWAN Class A and Class C devices by measuring average power consumption for varying payload sizes and spreading factors. The authors in [18] proposed a simple simulation model for estimating the autonomy of biomedical sensors in body sensor networks (BSN). The models consider different operating modes of MCU and radio.

A widespread technology that enables low-power WSNs is the BLE technology that is already available in a vast majority of modern smart devices (smart phones, tablets, smart watches, and computers). The omnipresence of BLE technology enables its quick adoption in consumer-oriented battery-powered low-power wireless devices. The authors in [19] presented BLE communication stack in detail including all communication layers, parameters, and stack behavior. They performed some performance evaluation tasks such as latency, energy consumption, and maximum piconet size and throughput using the CC2540 BLE radio chip. the authors in [9] proposed energy consumption models for opportunistic sensor data collection with BLE technology. They suggested two energy consumption models for different BLE approaches: an advertisement-based approach and connection-based approach. For each of the approaches, they measured average current consumption using the Bluegiga BLE121LR BLE platform. They derived analytical models by introducing BLE connectivity parameters and provided a detailed evaluation of proposed models considering the main BLE parameters. They derived the sensor node lifetime models for both cases, models for data collection efficiency in terms of energy consumed per collected data bit, and discussed the influence of bit error rate (BER) on all considered performance parameters. The authors in [10] conducted an in-depth analysis of BLE power consumption, particularly for estimation of discovery latency and energy consumption of both scanner and advertiser during a discovery process. The models were developed and validated for the BLE112 BLE chipset. If the user wants to use their models for other BLE chipsets, all platform-dependent parameters should be possible to adapt according to the selected BLE platform. They made the proposed model publicly available in the form of a software library. The authors in [20] presented their work with quantitative analysis for assessing the energy consumption of BLE advertisement procedures. The analysis includes a mathematical model for the device discovery dynamics and device performance evaluation under various BLE parameter settings. In [11,21], the authors focused on developing an analytical model and simulations for discovery probability, influence of parameter selection on discovery latency, and energy metrics of the discovery process.

### 2.2. Wireless ECG Sensor

**Figure 1:**
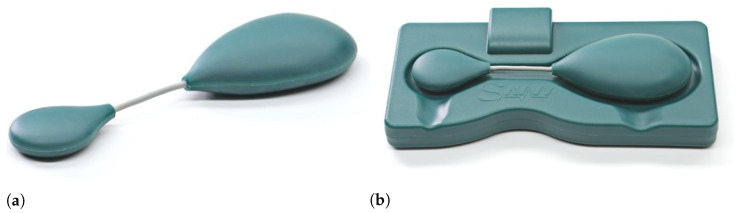
(**a**) Commercial version of the ECG body sensor. (**b**) The sensor placed on the charging dock.

The wireless ECG sensor investigated in this paper is certified as a Class IIa medical device and is intended for long-term continuous personal cardiac monitoring [4]. It has been available on the market since 2016 under the trademark Savvy ECG (http://savvy.si/, accessed on 29 April 2022). Physically, the sensor is small (dimensions: 130 × 35 × 14 mm) and light (weight: 21 g). It acts as a body gadget that can be worn on the skin by attaching it with two standard self-adhesive electrodes (Figure 1). Its outer shell is made of a waterproof and biocompatible plastic material. The device primarily measures differential surface electrical potential (ECG) between the proximal electrodes—a signal denoted as differential lead or differential ECG [22]. Experiments have shown that the optimal distance between the electrodes, which ensures satisfactory signal-to-noise ratio as well as minimal discomfort for the user, is around 8 cm [22] of flexible connection between the electrodes. This flexible part in combination with the snap fasteners that attach to the electrodes make the sensor adjustable for positioning and resilient to the movements of the user.

The hardware of the sensor implements the measurement functionality as follows: The first conversion of the measured quantity is made with an analog circuitry, including preamplifier and analog filters, that takes the input signal and converts it to appropriate voltage levels. Afterwards, the signal is passed to the MCU that converts the analog input signal into digital by sampling it with a predefined frequency and stores the samples into a memory buffer for further transfer. The samples are next transmitted in bulk by the MCU over a standard SPI (Serial Peripheral Interface) to the chip with a radio transceiver. The signal is then wirelessly transferred to a personal digital assistant (PDA), such as a smartphone or a tablet, by using a custom wireless protocol built on top of the BLE. The selected BLE radio for the sensor is the nRF8001 module by Nordic semiconductor, which supports Bluetooth 4.0 low-energy specifications. On the PDA, the data is stored and visualized in real time [23]. The ECG sensor represents a peripheral device in terms of BLE device classification, whereas the main device for ECG sample collection, i.e., the PDA, is called a central device.

The two electrical connectors of the sensor, additionally to connecting the electrodes to perform measurements, are also used to plug the sensor on a battery charger. A charger circuitry in the sensor detects when the battery charger is connected to the input snap terminals and puts the sensor in charging mode. Moreover, the battery charger is implemented as a charging dock designed to prevent reverse connection of the sensor on the charger. Additional components of the sensor, contributing to preserving sensor’s energy, are: the circuit breaker that isolates all the other circuits from the battery in order to minimize power consumption when the sensor is stored for a longer period of time and the power delivery circuitry used to lower the power usage while the sensor is not active by enabling the MCU to selectively deliver power to other building blocks.

As a compromise among sustainable power consumption, acceptable measurement quality, and acceptable bandwidth for most smartphones to handle, a moderate resolution of 128 samples/s is used and enforced by the software, but the hardware allows for it to be increased up to 1024 samples/s. The sensor has found its application in several areas in medicine and in every-day life [4]. It has been demonstrated that the moderate-resolution ECG is suitable for long-term personal cardiac activity monitoring, as well as for clinical use, such as screening patients with a suspicion of irregular heartbeat [13], prospective study of atrial fibrillation [24], and continuous remote monitoring of chronic obstructive pulmonary disease (COPD) patients [25]. Furthermore, the sensor has also been used for: abdominal fetal ECG monitoring, ECG monitoring in veterinary practice, on dogs, cats and horses, biometric authentication, and heart rate variability (HRV) biofeedback assessment. Its exceptionally lightweight design allows for unobtrusive use during sports activities or during exhaustive physical work as well [26]. In addition to ECG, it has been demonstrated that other features can also be extracted from the measured potential difference on the body’s surface, such as muscle activity and respiration [22,27]. Moreover, the standard 12-lead ECG can be synthesized from the measurements from three sensors placed in appropriate positions [28,29,30]. Lastly, the future addition of a temperature sensor and accelerometer [22] promises to transform the next generation of wearable sensors to a truly multifunctional design.

## 3. Sensor Operating Modes and Energy Consumption Measurements

The ECG sensor supports different operating modes to extend autonomy as far as possible. In this section, all operating modes are described in detail. The operating modes are later broken down to individual power consumption events. The individual power consumption events are measured and analyzed in order to identify the important parameters needed for power consumption modeling.

### 3.1. Operating Modes

The wireless ECG sensor supports several operating modes to comply with the power efficiency requirements imposed by its small size and battery operation:

- Shutdown mode
- Sleep mode
- Idle mode (disconnected mode): fast and slow advertising
- Active mode (ECG sampling).

In shutdown mode, all the electronic components on the wireless sensor board are disconnected from the battery for maximal power conservation, particularly for storing the device for longer periods of time. In particular, the device consumes fewer than 2 nA in shutdown mode, and this consumption is constant as there are no events in this mode of operation. The ECG sensor exits the shutdown mode when it is connected to the charging station.

When an ECG sensor is powered-on, it switches to idle mode, where it starts advertising itself by periodically transmitting BLE advertising packets. First, it starts in a fast advertising idle mode, where the BLE radio periodically transmits advertising packets. After the fast advertising period is over, the ECG sensor switches to slow advertising idle mode. The faster advertising rate during the fast advertising idle mode enables faster discovery of the ECG sensor by the central device (e.g., smartphone), while the slow advertising idle mode, on the other hand, enables a bit slower discovery of the ECG device while also reducing the power consumption, thus significantly prolonging the sensor’s autonomy.

The central device acts as a BLE scanner device that listens for a few seconds for connectable advertising packets containing the ECG sensor service identification; more than one second is typically required, but five seconds is advised to ensure that the ECG sensor can be discovered in its slow advertising mode. After the ECG sensor is discovered by the scanner, the central device initiates a standard nonencrypted BLE connection with the sensor. The ECG sensor then makes a transition to the active mode of operation: the ECG sensor is connected to the central device and actively streams ECG measurements.

Following a successful connection attempt, the central device issues several commands over a proprietary protocol defined on top of a client-writable GATT. GATT here stands for Generic Attribute Profile, which is a protocol most commonly used with the BLE. Among others, there are two commands that influence the sensor’s power consumption:

- Transmit (TX) power setting (options are 0 dBM, −6 dBM, −12 dBM, −18 dBM). The default in our case is 0 dBM.
- Sampling rate selection, which is limited to values that divide 32,768. The default in our case is 128 Hz.

If the ECG measurement is stopped by the user, then the central device triggers a disconnect. In such an event, the sensor stops sampling and switches to idle mode. In case the sensor battery is drained down to its minimum allowed voltage, the sensor disconnects and switches to shutdown mode, from which it can only be awakened by its battery charger. The last possibility is that the sensor moves out of the radio range and the connection on both devices times out. In this case, the sensor also stops sampling and switches to idle mode.

The focus in this paper is on the events consuming considerable amounts of energy, i.e., events in idle and active mode. Therefore, in the following, these operating modes are described in more detail regarding their power consumption profiles. First, we give details about the measuring equipment used to obtain the power consumption measurements.

### 3.2. Measurement Equipment

**Figure 2:**
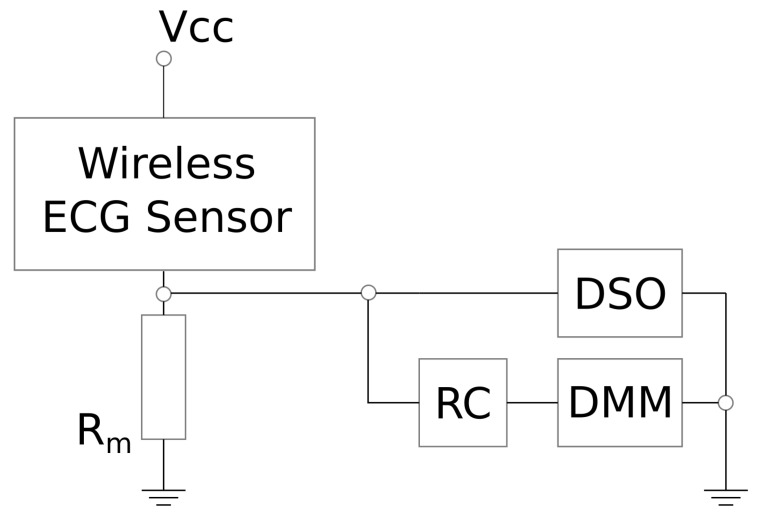
Schematic presentation of the current consumption measurement circuit.

To measure the power consumption of the wireless ECG sensor, we used the measurement circuit presented in Figure 2. The power consumption is measured indirectly by multiplying the measured current and battery voltage. The current is also measured indirectly by measuring the voltage on a shunt resistor with resistance of $R_{m}=10\;\Omega$. For the analysis of current consumption during individual detectable events, a digital sampling oscilloscope (DSO) Rigol DS1054 is used. With the DSO, we recorded a current consumption envelope with high resolution, which enabled the separation of fine details in the current consumption needed to define the analytical power consumption models. We measured the difference between the start of an event and the end of the event on the current profile in order to assess the duration parameter of the event. The DSO resolution was very high—50 million samples per second, which is equal to a time resolution of 20 nanoseconds.

For average power consumption analysis, we used the HP3478A benchtop digital multimeter (DMM) with Resistor-Capacitor (RC) low-pass filter (resistance $R_{f}=10\;k\Omega$ and capacitance $C_{f}=1000\;\mu F$). The RC filter improves the averaging by suppressing high-frequency components in the measurement signal to prevent aliasing from the limited DMM sampling frequency. According to the Nyquist–Shannon theorem, the sampling frequency should be at least twice as high than the highest frequency component of the measured signal in order to guarantee perfect reconstruction of the sampled (measured) signal. Therefore, perfect signal reconstruction is possible for a signal with a bandwidth *B* lower than half of the sampling frequency $f_{s}$ ($B<f_{s}/2$).

Additionally, to obtain measurements for different TX power settings and sampling rates, a Linux-based single-board computer (SBC) with BLE capability was used. The SBC acts as a central device and exposes the options to set a desired connection interval as well as a desired sampling rate.

### 3.3. Consumption Profiles in Active and Idle Mode

**Figure 3:**
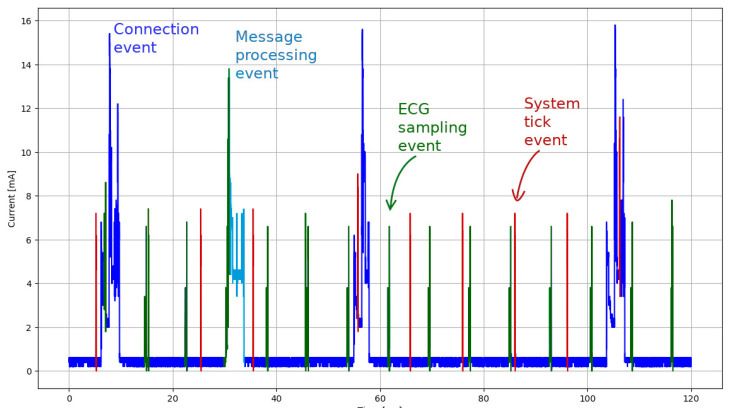
Current consumption profile recording in an active mode (i.e., while streaming ECG samples).

Two types of communication events are defined in the BLE specification: advertising events and connection events. Figure 3 presents a short excerpt from one current recording representing the active mode where individual activity events can be identified: connection event, message-processing event, ECG sampling event, and system tick event. Among all the detected events, both MCU and BLE radio are in *sleep mode*, with both of them consuming almost no energy at all. Low current spikes near the noise floor represent the MCU activity bursts. In the following subsections, we describe all possible energy consumption modes that can be identified from the current profile recordings. Additionally to the active mode events, the consumption measurements for the advertising event in idle mode and in the sleep mode are also described.

#### 3.3.1. Connection Event

**Figure 4:**
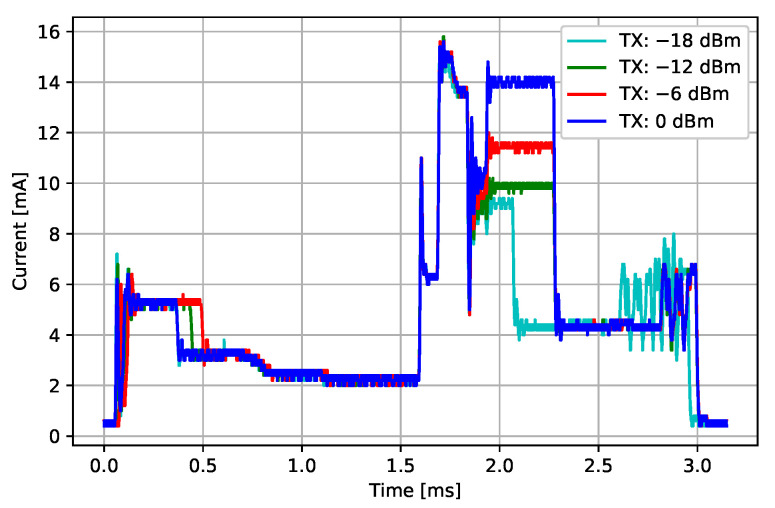
Current consumption profiles of BLE connection events for different TX power settings.

During the BLE connection event, devices exchange all the data that was prepared during the last time between two consecutive connection events. The number of messages exchanged during one connection interval can differ, but for the given ECG sensor, most connection events exchange the same amount of data. One example of a BLE connection event current consumption profile recording is presented in Figure 4. The current profile starts by the BLE radio wake-up procedure between 0.0 ms and 1.5 ms, followed by an RX event from 1.5 ms to approximately 1.8 ms. Then, a TX event follows, which lasts until approximately 2.75 ms, continuing with the BLE stack processing, and ending with the transition of the BLE radio back into sleep mode.

Figure 4 shows current profiles for 4 different TX power settings: 0 dBm, −6 dBm, −12 dBm and −18 dBm. We can observe that the energy consumption during a connection event for different TX power settings is different, which enables the designers to extend the autonomy of the sensor in closer operating ranges by reducing the transmitter power settings. The results of the energy consumption measurements for different TX power settings are presented in Table 1.

#### 3.3.2. Message-Processing Event

**Figure 5:**
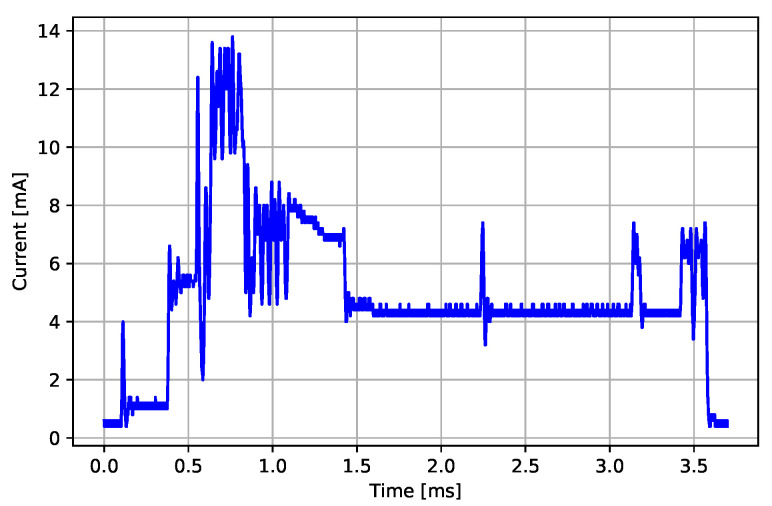
Current consumption profile of a BLE message-processing event.

Each time an ECG packet buffer in the MCU becomes full, the MCU transfers the Application Controller Interface (ACI) packet to the radio for transmission. The message-processing event looks very similar to a BLE connection event, except there is no RF communication involved during the event. A communication controller inside the BLE radio processes the incoming ACI packet and updates the on-board BLE services accordingly to prepare for information exchange during the next connection event. The transmitter and the receiver stay in an idle mode during the ACI packet processing event. All the activity in the nRF8001 radio is concerned with the BLE stack message processing and preparation for transmission during the next nearest BLE connection event. The measured mean message-processing event energy consumption $e_{msg}$ is 79.21 μJ with 0.64 μJ standard deviation. An example of a message-processing event’s current consumption profile is presented in Figure 5. The BLE message-processing event begins with the BLE radio wake-up procedure seen from 0.0 ms to about 0.3 ms, followed by a BLE stack processing between 0.3 ms and 3.5 ms, and ends with transition of the BLE radio back to sleep mode.

#### 3.3.3. ECG Sampling Event

**Figure 6:**
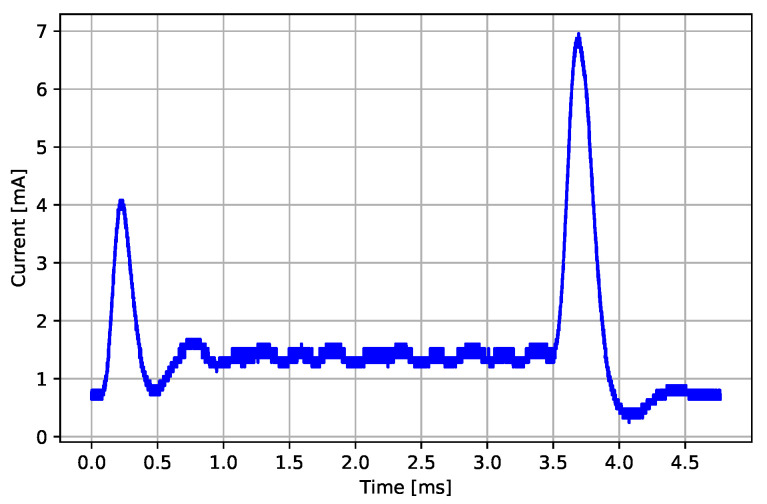
Current consumption profile of an MCU ECG sampling event.

The ECG sampling is performed in equidistant ECG sampling events. The ECG sensor uses a 32.768 kHz RTC (real-time clock) quartz oscillator as a high-precision reference clock to minimize the jitter. The internal timer is set to regular interrupt intervals in accordance with the ECG sampling frequency. At each interrupt, the MCU is awakened from sleep mode and analog-to-digital (A/D) conversion is started. When the A/D conversion is finished, the ADC triggers an interrupt routine, where a fresh A/D sample is added to the existing ECG packet buffer, and then the MCU returns to sleep if the buffer is not full. If the buffer is full, then MCU transfers the ECG packet buffer to the radio for transmission before returning to sleep. An example of an ECG sampling event is presented in Figure 6. The ECG sampling event begins with the wake-up triggered by the ECG sampling timer, followed by the ECG sampling procedure, and ends with the processing of the ECG sample and the transition back to sleep mode.

The energy consumption of an ECG sampling event $e_{sample}$ is calculated as a sum of current consumption profile samples $i_{t}$ multiplied by a sampling interval Δt and battery voltage $V_{batt}$ (Equation (1)). The measured mean energy consumption per ECG sampling event is 2.78 μJ with 0.03 μJ standard deviation.

(1) $$e_{sample}=V_{batt}\sum_{t=0}^{T}i_{t}\times \Delta t$$

#### 3.3.4. System Tick Event

**Figure 7:**
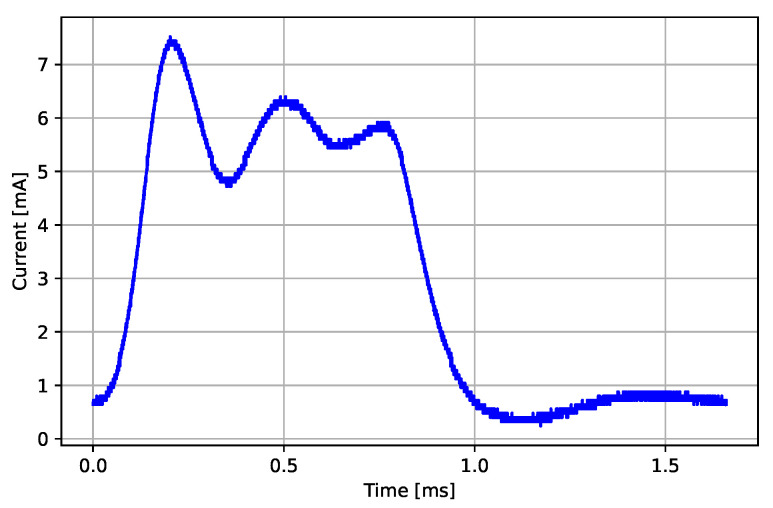
Current consumption profile of an MCU tick event.

The MCU uses a system tick timer for real-time event execution. A tick timer is set for continuous operation with a predefined time interval based on a clock from the 32.768 kHz RTC quartz oscillator. Each time the tick interrupt is generated, the MCU increases the tick counter and decreases the remaining time of tasks waiting for execution. The measured mean energy consumption $e_{tick}$ of the MCU system tick event with a period of $t_{tick}=0.133\;ms$ is 1.85 μJ with 0.0 μJ standard deviation. An example of a current consumption profile associated with an MCU system tick event is presented in Figure 7. The current profile is actually a short burst of activity where the MCU wakes up after being interrupted by the system tick timer, increases the value of the system tick counter, and returns to sleep.

#### 3.3.5. BLE Advertising Event

**Figure 8:**
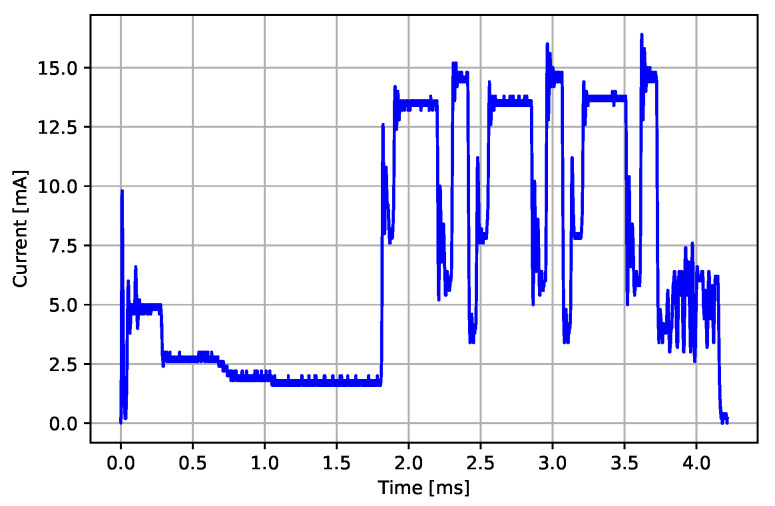
Current consumption profile of an advertising event.

During one BLE advertising event, the BLE radio transmits the advertising packet on three consecutive advertising channels and listens for a short period on each channel for possible requests for connection from a scanner device (initiator), which searches for the available ECG sensors. We averaged the current consumption profiles for 10 advertising events to suppress the effects of slight deviations between them. The energy consumption of a BLE advertising event $e_{adv}$ can be calculated in the same way as for the ECG sampling event—as a sum of all current consumption samples $i_{t}$ multiplied by a sample time Δt and battery voltage $V_{batt}=4.13\;V$ (Equation (2)). The average measured advertising energy consumption is 120.65 μJ with 2.43 μJ standard deviation. The resulting power consumption profile is presented in Figure 8. The power (current) consumption profile begins with a radio wake-up event ranging from 0.0 ms to approximately 1.8 ms, and the following pattern from approximately 1.8 ms to approximately 3.7 ms represents three consecutive RX and TX events on three different advertising channels. The last part of the BLE advertising event represents the BLE stack processing and the transition to the radio sleep state.

(2) $$e_{adv}=V_{batt}\sum_{t=0}^{T}i_{t}\times \Delta t$$

#### 3.3.6. Sleep Mode

Among all the events, both the BLE radio and the MCU wait in either idle or sleep mode, which are both power-saving modes. According to the official documentation, the nRF8001 BLE radio consumes 2 μA in idle mode between connection or advertising events [31]. The MSP430F2274 MCU consumes only around 1 μA in low-power mode 3 (LPM3), from where very quick transitions to an active mode are possible (fewer than 1 μs) [32]. The measured ECG sensor’s sleep current consumption in active mode $I_{sleep\_active}$ is 716 μA, which translates to $P_{sleep\_active}$ = 2.957 mW at $V_{batt}$ = 4.13 V battery voltage. During the ECG sensor’s idle mode, the sleep current consumption $I_{sleep\_idle}$ is 265 μA, which translates to $P_{sleep\_idle}$ = 1.095 mW at $V_{batt}$ = 4.13 V battery voltage. This measurement includes the current consumption of the entire sensor board with all analog circuits included.

## 4. Power Consumption Models

The proposed power consumption models describe two main operating modes of the sensor: the idle mode and the active mode. In the idle mode, the sensor sends advertisement packets at regular intervals to be discoverable by the PDAs for data acquisition. Active mode, on the other hand, acts as a data collection mode where the MCU collects ECG samples and forwards them to the BLE radio for transmission to the connected PDA. The models are based on the parameters identified in the previous section, i.e, measured during the events that constitute a certain mode, idle or active. The events are, however, standard from the perspective of BLE specification. Different hardware and software implementations of BLE transceivers can have different event power profiles, but the events are equal from the protocol perspective. The only requirement for another BLE sensor platform to use the proposed energy consumption model is to measure the energy consumption for the identified events previously.

### 4.1. Idle Mode Power Consumption Model

The proposed analytical power consumption model for the idle mode ${\bar{P}}_{adv}$ is presented in a mathematical form with Equation (3). $T_{adv}$ is the advertising period for a selected advertising mode: $T_{adv\_fast}$ for the fast advertising and $T_{adv\_slow}$ for the slow advertising. $E_{adv}$ is the total energy consumed during advertising events, $E_{tick}$ is the total amount of energy consumed during system tick events, and $P_{sleep}$ is the power consumption during sleep mode. $T_{sleep}$ is the total sleep time of the ECG sensor during a $T_{adv}$ period. $e_{adv}$ and $e_{tick}$ represent the energy consumption of the individual advertising and system tick events, respectively, while $tp_{adv}$ and $tp_{tick}$ are the periods of the advertising and system tick events, respectively. $t_{adv}$ and $t_{tick}$ are the duration of the advertising event and the system tick event, respectively.

(3) $$\begin{array}{cc} {\bar{P}}_{adv} & =\frac{1}{T_{adv}}\left(E_{adv}+E_{tick}\right)+\frac{1}{T_{adv}}P_{sleep\_idle}T_{sleep}\\ \; & =\frac{1}{T_{adv}}\left(\frac{T_{adv}}{tp_{adv}}e_{adv}+\frac{T_{adv}}{tp_{tick}}e_{tick}\right)+\frac{1}{T_{adv}}P_{sleep\_idle}T_{adv}\left(1-\frac{t_{adv}}{tp_{adv}}-\frac{t_{tick}}{tp_{tick}}\right)\\ \; & =\frac{e_{adv}}{tp_{adv}}+\frac{e_{tick}}{tp_{tick}}+P_{sleep\_idle}\left(1-\frac{t_{adv}}{tp_{adv}}-\frac{t_{tick}}{tp_{tick}}\right) \end{array}$$

This model gives us the average power consumption during a selected idle mode setting: fast advertising or slow advertising. If we want to calculate the overall average power consumption ${\bar{P}}_{idle}$ in the idle mode (combining fast and slow advertising), Equation (4) should be used. $T_{adv\_slow}$ and $T_{adv\_fast}$ are slow advertising and fast advertising periods, respectively, where the average power consumption for slow advertising ${\bar{P}}_{adv\_slow}$ and the average power consumption for fast advertising ${\bar{P}}_{adv\_fast}$ can be calculated using Equation (3).

(4) $${\bar{P}}_{idle}=\frac{T_{adv\_slow}{\bar{P}}_{adv\_slow}+T_{adv\_fast}{\bar{P}}_{adv\_fast}}{T_{adv\_slow}+T_{adv\_fast}}$$

### 4.2. Active Mode Power Consumption Model

In the active mode, the ECG sensor connects to the controlling device, such as a mobile phone, and starts sampling and streaming the ECG signal. The average power consumed during the active mode ${\bar{P}}_{conn}$ is the sum of all energy consumed in active mode divided by the time period *T* which we are measuring the average power consumption for. The total energy consumed consists of the energy consumed in connection events $E_{conn}$, ECG sampling events $E_{sample}$, system tick events $E_{tick}$, and sleep mode $P_{sleep\_active}\times T_{sleep}$. We can calculate the total sleep time $T_{sleep}$ as the difference between the total time period *T* and the sum of time spent in all other events.

The proposed analytical model for the power consumption in active mode is presented with Equation (5), where $e_{conn}$ is the energy consumed during one connection event, $e_{msg}$ is the energy consumed during one message-processing event, $e_{sample}$ is the energy consumed during an ECG sampling event, $e_{tick}$ is the energy consumed during one system tick event, $tp_{conn}$ is the connection interval, $tp_{msg}=14\times tp_{sample}$ is the message-processing interval that happens at every 14th ECG sampling event (assuming that each ECG packet contains 14 ECG samples), $tp_{sample}$ is the ECG sampling interval, and $tp_{tick}$ is the system tick interval. $t_{conn}$, $t_{msg}$, $t_{sample}$, and $t_{tick}$ are the duration of the connection event, the message-processing event, the ECG sampling event, and the system tick event, respectively. Entering actual connection parameters into Equation (5) gives an estimation of the power consumption in an active mode, which is the basis for the sensor autonomy estimation.

(5) $$\begin{array}{cc} {\bar{P}}_{conn} & =\frac{1}{T}\left(E_{conn}+E_{sample}+E_{tick}\right)+\frac{1}{T}P_{sleep\_active}T_{sleep}\\ \; & =\frac{1}{T}\left(\frac{T}{tp_{conn}}e_{conn}+\frac{T}{tp_{msg}}e_{msg}+\frac{T}{tp_{sample}}e_{sample}+\frac{T}{tp_{tick}}e_{tick}\right)\\ \; & +\frac{1}{T}P_{sleep\_active}T\left(1-\frac{t_{conn}}{tp_{conn}}-\frac{t_{msg}}{tp_{msg}}-\frac{t_{sample}}{tp_{sample}}-\frac{t_{tick}}{tp_{tick}}\right)\\ \; & =\frac{e_{conn}}{tp_{conn}}+\frac{e_{msg}}{tp_{msg}}+\frac{e_{sample}}{tp_{sample}}+\frac{e_{tick}}{tp_{tick}}+P_{sleep\_active}\left(1-\frac{t_{conn}}{tp_{conn}}-\frac{t_{msg}}{tp_{msg}}-\frac{t_{sample}}{tp_{sample}}-\frac{t_{tick}}{tp_{tick}}\right) \end{array}$$

## 5. Validation of the Models and Power Consumption Analysis

The proposed power consumption models from Section 4 and measured energy consumption for all the separate activity events from Section 3 are used for the validation of models and power consumption analysis for different connection settings.

### 5.1. Idle Mode Power Consumption

All measured idle mode power consumption parameters presented in Section 3 are summarized in Table 2. The ECG sensor cannot be put in fast advertising and slow advertising modes manually. It alternates between the two advertising modes automatically, and therefore the measurements of individual advertising modes have to be differentiated based on the readings of the multimeter. Both advertising periods are long enough for the values on the multimeter to settle, and reliable current values can be read. The overall average advertising current consumption $P_{adv\_avg}$ can be calculated from both of the measured current consumptions—$P_{adv\_fast}$ and $P_{adv\_slow}$—as presented with Equation (4).

In Table 3, both measured values and model-based values for the idle mode of the ECG sensor are shown.The $P_{measured}$ and $I_{measured}$ values represent the measured power and current consumption, respectively, while $P_{model}$ and $I_{model}$ represent the model-based calculated power and current consumption values, respectively. The results show that the measured and calculated values are very similar, which presents a very good model fit to the actual power and current consumption values, with less than 2% maximal error in current consumption, while average advertising current consumption fits perfectly. The error is calculated as deviation of the modeled value from the measured value, and expressed as percentage of the measured value.

### 5.2. Active Mode Power Consumption

For the active mode power consumption analysis, we selected 12 possible settings: all combinations of 4 TX power settings and 3 ECG sampling rates measured in samples per second (sps). All measured model parameters for power consumption estimation of the 12 settings are summarized in Table 4. Table 5 presents both measured and model-based power and current values for the active mode. The results show that the model-based values and the measured values are very close for all settings with the lowest sampling rate (128 sps). In the case of higher sampling rates, the model overestimates the current consumption.

### 5.3. Analysis of Sensor Autonomy

To translate power consumption to sensor autonomy, we divide the battery capacity by the average current consumption in the selected sensor mode. The analyzed sensor has an integrated 240 mAh lithium-ion battery that stores enough energy for several days of continuous ECG signal streaming. The results of ECG sensor autonomy estimation based on measured average current consumption and estimated current consumption are collected in Table 6. The results show that by quadrupling the ECG sampling rate and thus quadrupling the amount of bytes transferred, the sensor autonomy is reduced by a factor of two. The typical autonomy of modern smartphones, assuming an average user, is around one day, while the sensor on a single charge lasts for around four days with 512 sps and more than eight days with 128 sps. When the sensor is neither in active mode nor in shutdown mode, it stays in idle mode, where advertising takes place with average advertising power consumption $P_{adv\_avg}=1.308\;mW$, which gives us 31.5 days of autonomy while waiting to start ECG measurements. Thus, with regular weekly use, shutting down the sensor seems unnecessary. This further simplifies the usage for non-tech-savvy users.

The most influencing parameter on the sensor autonomy is the ECG sampling rate. Sampling rate directly imposes requirements on the length of connection interval, which has to be, on one hand, short enough to enable transfer of all recorded ECG data, while, on the other hand, long enough not to waste energy with unnecessary empty connection events. By decreasing the length of the connection interval, the density of transmission events increases, which consequently increases the average power consumption. The optimal connection interval for the selected sampling interval $tp_{sample}$ is therefore in our case $tp_{conn}=14\times tp_{sample}$, where 14 samples are packed in a single ECG report packet.

Before providing guidelines for further development of the ECG sensor, additionally to taking into consideration the power anatomy analysis, we also consider the experience gathered from users who bought/rented the device for themselves [12] or used the device in a renting scheme with weekly turn-around [13]. While most of the experience is beyond the scope of this article, the most important findings are summarized below. The first finding refers to the current autonomy of the sensor as more than sufficient. While the sensor has been mostly used with maximum transmission power and a very low sampling frequency of 128 Hz, its autonomy far surpassed both the autonomy of smartphones, which it was used in combination with, and the period in which people felt comfortable wearing it continuously. While the latter varies from person to person, most people are inclined to remove it after 2 days of use or less. This is not due to the weight of the device worn on one’s chest—most people find it completely unobtrusive—but because self-adhesive electrodes are required, which are prone to irritating the skin. Therefore, the autonomy could be reduced to just above 2 days under some usage scenarios. The second finding was that the sensor’s sampling frequency of 128 samples/s was often too low for clinical use. The medical personnel examining measurements expressed their wish for more details, which would broaden the specter of potential use of the sensor. Since the sensor is an excellent platform for long-term heart rate variability monitoring, the rate of 250–500 samples/s was taken as a design goal, based on research which excluded lower frequencies as insufficient [33]. Lastly, the processing requirements of the sensor have not increased, so neither the MCU replacement nor a change in its usage pattern was planned.

Having the power consumption model available, as well as the above experience, both user-subjective and medical, we are able to make recommendations regarding the sensor redesign. Our recommendations are to increase the sampling frequency to 512 Hz and leave radio transmission power at 0 dB, since its effect on autonomy is insignificant. The sampling frequency increase is required to increase the sensor’s capabilities, often required by the medical personnel, and is enabled by the continuous development of more capable smartphone hardware, which no longer struggle with receiving more than 128 samples per second. Furthermore, we envision two scenarios regarding the battery capacity, which can be used to tailor the next version of the sensor. The first scenario is to decrease the battery capacity to 160 mAh, provided that it would also lead to sensor size, weight, and price reduction. While a smaller battery would primarily increase comfort, it might also help reduce the motion-induced measurement artifacts due to reduced sensor weight. Under this scenario, the users would be required to recharge the sensor every 2 days or less, which would still be acceptable since a large portion of users already require a break from the irritable self-adhesive electrodes in nearly the same time. In the second scenario, the battery could be increased to 400 mAh, extending the autonomy to over 5 days at 512 Hz sampling rate, which might be seen as beneficial for health care services where the sensor is lent to a different user every week. The users would therefore not even need a charger, which would make this scenario more user-friendly. The battery would have to be larger and heavier though, which might create some unforeseen adverse effects for the engineers designing the device and for the users wearing it. Given that, so far, no user has complained about the device weight, this might be a path worth exploring.

## 6. Conclusions

In this paper, we thoroughly investigated the sources of power consumption in a modern wireless ECG sensor and proposed analytical power consumption models for the most important activity modes. We designed a model for active ECG streaming mode, which slightly overshoots the measured average current consumption for higher ECG sampling rates, but nevertheless serves as a good predictor of the wireless sensor autonomy.

The limitation of the presented approach is that for each new platform which we want to estimate power consumption for, we need to make measurements of all the parameters used in the proposed models. This arises from the variability of the platforms available on the market for BLE sensors. Different BLE modules may have different base power consumption due to different implementations of the communication stack. The central processing unit (CPU) used for the application can also have a large impact on the power consumption, depending on the CPU performance and the complexity of the application running on it. The power consumption estimates can be used for similar platforms, but without the measurement of base current consumption events, significant deviations from the results presented in this manuscript are to be expected.

Based on the presented results, we can conclude that it is not possible to make a power consumption model for the general use of BLE devices. There are some useful guidelines though regarding the manner the current consumption of BLE devices can be tamed. The results show that the transmit power has a negligible effect on the sensor autonomy in the case of streaming data with high sampling rates. The most energy can be saved by lowering the sampling rate and by packing as much data as possible in a single BLE packet. Finally, selecting a suitable connection interval for the applied sampling rate can have the strongest energy-saving effect.

Further optimizing such a sensor is a difficult task. We propose two scenarios in which the battery could be either increased or decreased according to current usage patterns. Apart from the battery capacity, other sensor hardware and software parameters cannot be optimized for significant power consumption reduction.

## Figures and Tables

**Table 1:** Energy consumption of BLE connection events for different TX power settings.

TX Power Setting [dBm]	$e_{\mathrm{conn}}$ [μJ]	std$\left(e_{\mathrm{conn}}\right)$ [μJ]
0	73.01	0.65
−6	68.92	0.77
−12	66.50	1.00
−18	65.48	0.47

**Table 2:** BLE idle mode evaluation parameters.

Parameter	Value
$T_{adv\_slow}$	300 s
$T_{adv\_fast}$	30 s
$tp_{adv\_slow}$	1000 ms
$tp_{adv\_fast}$	100 ms
$tp_{tick}$	1000 ms
$t_{adv}$	4.038 ms
$t_{tick}$	0.133 ms
$P_{sleep\_idle}$	1.095 mW
$e_{adv}$	120.65 μJ
$e_{tick}$	1.85 μJ

**Table 3:** Comparison of measured and model-based power and current consumption for idle mode. Model error is presented as percentage of the measured value.

Mode	$P_{\mathrm{model}}$ [mW]	$I_{\mathrm{model}}$ [mA]	$P_{\mathrm{measured}}$ [mW]	$I_{\mathrm{measured}}$ [mA]	Error [%]
Slow Advertising	1.213	0.294	1.210	0.293	+0.34
Fast Advertising	2.259	0.547	2.292	0.555	−1.44
Advertising	1.308	0.317	1.308	0.317	0.00

**Table 4:** BLE active mode evaluation parameters.

Parameter	Sampling Rate = 128 sps	Sampling Rate = 256 sps	Sampling Rate = 512 sps
$tp_{conn}$	80.0 m s	36.0 m s	14.0 m s
$tp_{sample}$	7.8 m s	3.9 m s	2 ms
$tp_{msg}$	109.2 m s	54.6 m s	28 ms
$tp_{tick}$	10 ms	10 ms	10 ms
$t_{conn}$	2.987 m s	2.987 m s	2.987 m s
$t_{msg}$	3.543 m s	3.543 m s	3.543 m s
$t_{sample}$	0.373 m s	0.373 m s	0.373 m s
$t_{tick}$	0.133 m s	0.133 m s	0.133 m s
$P_{sleep\_active}$	2.980 m W	2.980 m W	2.980 m W
$e_{conn\_0d\mathrm{Bm}}$	73.13 μ J	73.13 μ J	73.13 μ J
$e_{conn\_-6d\mathrm{Bm}}$	69.06 μ J	69.06 μ J	69.06 μ J
$e_{conn\_-12d\mathrm{Bm}}$	66.63 μ J	66.63 μ J	66.63 μ J
$e_{conn\_-18d\mathrm{Bm}}$	65.60 μ J	65.60 μ J	65.60 μ J
$e_{msg}$	79.35 μ J	79.35 μ J	79.35 μ J
$e_{sample}$	2.78 μ J	2.78 μ J	2.78 μ J
$e_{tick}$	1.85 μ J	1.85 μ J	1.85 μ J

**Table 5:** Comparison of measured and model-based power and current consumption for active mode. Model error is expressed as percentage of the measured value.

TX [dBm]	Sampling [sps]	$P_{\mathrm{model}}$ [mW]	$I_{\mathrm{model}}$ [mA]	$P_{\mathrm{measured}}$ [mW]	$I_{\mathrm{measured}}$ [mA]	Error [%]
0	128	4.752	1.151	4.774	1.156	−0.43
	256	6.580	1.593	6.393	1.548	+2.91
	512	10.994	2.662	10.123	2.451	+8.61
−6	128	4.701	1.138	4.737	1.147	−0.78
	256	6.467	1.566	6.311	1.528	+1.83
	512	10.703	2.592	9.932	2.405	+7.78
−12	128	4.671	1.131	4.712	1.141	−0.88
	256	6.400	1.550	6.282	1.521	+1.91
	512	10.529	2.549	9.809	2.375	+7.33
−18	128	4.658	1.128	4.700	1.138	−0.88
	256	6.371	1.543	6.245	1.512	+2.05
	512	10.456	2.532	9.767	2.365	+7.06

**Table 6:** ECG sensor autonomy estimation based on measured average current consumption and estimated model-based current consumption. Model error is expressed as percentage of the measured value.

TX [dBm]	Sampling [sps]	Autonomy $\left(I_{\mathrm{measured}}\right)$ [days]	Autonomy $\left(I_{\mathrm{model}}\right)$ [days]	Error [%]
0	128	8.65	8.69	+0.46
	256	6.46	6.28	−2.79
	512	4.08	3.76	−7.84
−6	128	8.72	8.79	+0.80
	256	6.54	6.39	−2.29
	512	4.15	3.86	−6.99
−12	128	8.76	8.84	+0.91
	256	6.57	6.45	−1.82
	512	4.21	3.92	−6.89
−18	128	8.79	8.87	+0.91
	256	6.61	6.48	−1.97
	512	4.23	3.95	−6.62

## Data Availability

Not applicable.

## Abbreviations

The following abbreviations are used in this manuscript:

ACI	Application Controller Interface
ADC	Analog-to-digital converter
A/D	Analog-to-digital
BLE	Bluetooth Low Energy
CDCA	Coordinated duty cycle algorithm
COPD	Chronic obstructive pulmonary disease
CPU	Central processing unit
DC	Direct current
DMM	Digital multimeter
DSO	Digital sampling oscilloscope
ECG	Electrocardiogram
FDMA	Frequency-division multiple access
GATT	Generic attribute
IEEE	Institute of Electrical and Electronics Engineers
ISM	Industrial, scientific, and medical
LR-WPAN	Low-rate wireless personal area network
MCU	Microcontroller unit
PCB	Printed circuit board
PDA	Personal digital assistant
PHY	Physical layer
RC	Resistor-Capacitor
RF	Radio frequency
RTC	Real-time clock
RX	Receive
SBC	Single board computer
SIG	Special Interest Group
SPI	Serial peripheral interface
sps	samples per second
TDMA	Time-division multiple access
TX	Transmit
WBAN	Wireless body area network
WPAN	Wireless personal area network
WSN	Wireless sensor network
